# Clinical results of proton beam therapy for twenty older patients with esophageal cancer

**DOI:** 10.1515/raon-2015-0034

**Published:** 2015-11-27

**Authors:** Takashi Ono, Tatsuya Nakamura, Yusuke Azami, Hisashi Yamaguchi, Yuichiro Hayashi, Motohisa Suzuki, Yoshiomi Hatayama, Iwao Tsukiyama, Masato Hareyama, Yasuhiro Kikuchi, Kenji Nemoto

**Affiliations:** 1 Department of Radiation Oncology, Southern Tohoku Proton Therapy Center, Koriyama, Fukushima, Japan; 2 Department of Radiation Oncology, Yamagata University Faculty of Medicine, Yamagata, Japan

**Keywords:** proton therapy, aged, esophageal neoplasms, radiotherapy

## Abstract

**Background:**

In an aging society, increasing number of older patients are diagnosed with esophageal cancer. The purpose of this study was to assess the clinical efficacy and safety of proton beam therapy for older patients with esophageal cancer.

**Patients and methods.:**

Older patients (age: ≥ 65 years) newly diagnosed with esophageal cancer between January 2009 and June 2013 were enrolled in this study. All patients underwent either proton beam therapy alone or proton beam therapy with initial X-ray irradiation. Toxicities were evaluated using the Common Terminology Criteria for Adverse Events version 4.0.

**Results:**

Twenty patients were eligible for this study and all completed the treatment. The median age was 78 years (range: 65–89 years) and the median follow-up time was 26.5 months (range: 6–62 months). Seven patients had lymph node metastases and 10 had stage II/III cancer. The median dose of proton beam therapy was 72.6 Gy relative biological dose effectiveness (RBE) (range: 66–74.8 Gy [RBE]) for proton beam therapy alone and 33 Gy (RBE) (range: 30.8–39.6 Gy [RBE]; total dose range: 66.8–75.6 Gy [RBE]) for proton beam therapy with initial X-ray irradiation. The 2-year overall survival rate was 81.8% (95% confidence interval [CI]: 62.4%–100%), and the 2-year local control rate was 89.4% (95% CI: 75.5%–100%). Grade 2 or 3 toxicities occurred in some cases; however, no grade 4 or 5 toxicity was observed.

**Conclusions:**

High-dose (66–75.6 Gy [RBE]) proton beam therapy without chemotherapy was an efficacious and safe treatment for older patients with esophageal cancer.

## Introduction

Esophageal cancer is the sixth leading cause of cancer death and the eighth most common cancer worldwide.^1^ In eastern Asia, esophageal cancer is the fourth most common cause of cancer death.^2^ Surgery remains the main treatment choice for resectable esophageal cancer. However, following reports from the Radiation Therapy Oncology Group^3^,^4^ and studies of the efficacy of chemoradiotherapy (CRT)^5^–^7^, CRT has become another choice for the treatment of esophageal cancer.

In an aging society, an increasing number of older patients are diagnosed with esophageal cancer. Not all of these patients can be treated with CRT or surgery because of their age, general condition and/or complications, although there are some reports regarding the use of CRT or surgery in older patients with esophageal cancer.^8^,^9^ Other studies have reported the efficacy of radiotherapy alone for older patients.^9^,^10^ However, compared with CRT or surgery, X-ray irradiation alone has not shown satisfactory results for the treatment of esophageal cancer.^3^,^9^

New radiotherapy treatments, such as intensity-modulated radiotherapy and proton beam therapy (PBT), deliver concentrated doses to the target volume, avoiding the organs at risk.^11^–^13^ These therapies may thus be suitable for treating older patients with esophageal cancer. Despite the increased use of PBT for esophageal cancer^14^–^18^, few data are available regarding the efficacy of PBT in older patients with esophageal cancer. In this study, we treated older patients with esophageal cancer using PBT without chemotherapy. We retrospectively evaluated the efficacy and safety of PBT in these older patients.

## Patients and methods

### Patients

Patients newly diagnosed with esophageal cancer treated with PBT without chemotherapy between January 2009 and June 2013 at the Southern Tohoku Proton Therapy Center were recruited from our database retrospectively. All patients were histologically confirmed to have esophageal cancer based on a biopsy before each treatment. Every patient was assessed, and the clinical stage of esophageal cancer was determined using endoscopy, computed tomography (CT) and positron emission tomography (PET)-CT. Written informed consent was obtained from all patients and the investigators followed recommendations of the Helsinki Declaration.

The inclusion criteria were as follows: a histologically confirmed diagnosis of esophageal cancer, age of ≥ 65 years, World Health Organization performance status of 0–2 and no distant organ metastasis or other sites of uncontrolled cancer.

### Proton beam therapy

**FIGURE 1.**
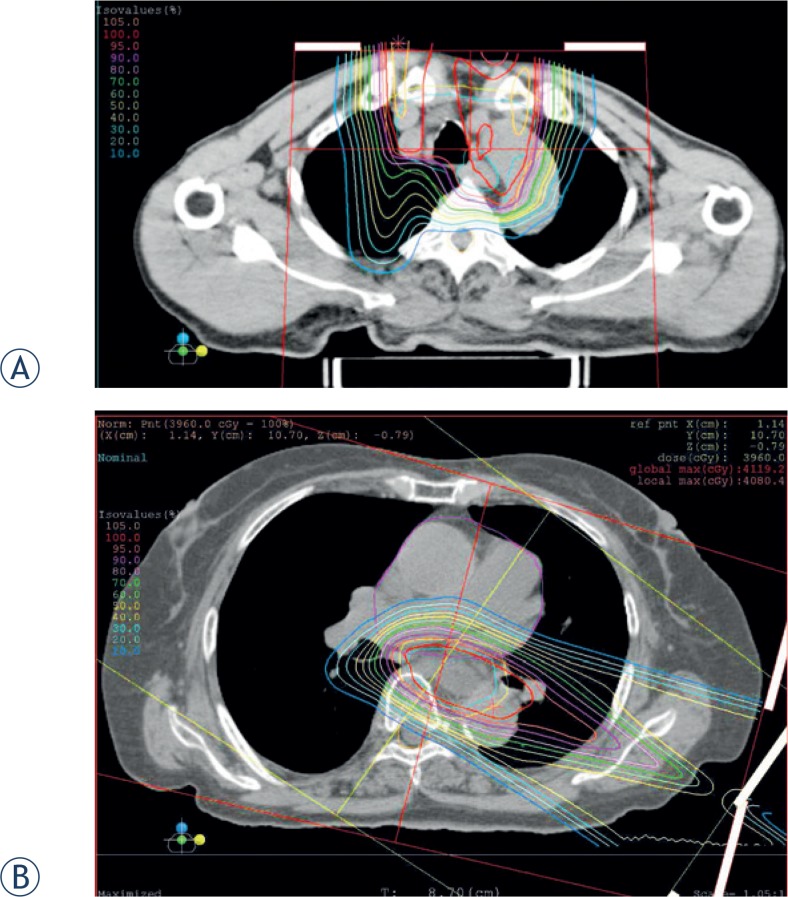
Dose distribution map for proton beam therapy following initial X-ray irradiation. The region outside the outermost line received <10% radiation. **(A)** Dose distribution map for cephalic esophageal cancer. **(B)** Dose distribution map for thoracic esophageal cancer.

Treatment planning for PBT was based on three-dimensional CT images taken at 2 mm intervals in the exhalation phase while using a respiratory gating system (Anzai Medical, Tokyo, Japan). The gross tumor volume (GTV) included the primary tumor and lymph node metastases. The primary tumor volume was determined from markers implanted using endoscopy at the cranial and caudal ends of the tumor. Lymph nodes over 1.0 cm in the short axis or exhibiting a high ^18^F-fluorodeoxyglucose uptake on PET-CT were considered metastases. The clinical target volume (CTV) was defined as GTV plus longitudinal margins of ≥ 2.5 cm and lateral margins of 0.5 cm. The planning target volume (PTV) was CTV plus 0.5 cm margins. The daily PBT fraction was 2.2 Gy relative biological dose effectiveness (RBE). Proton energy levels of 150 MeV or 210 MeV for 1–2 portals, and spread-out Bragg peak were tuned as much as possible until the PTV was exposed to a 90% isodose of the prescribed dose (Figure 1). The PBT system at our institute (Proton beam system, Mitsubishi, Tokyo, Japan) used synchrotron, and scattering methods. Treatment was administered during the exhalation phase using a respiratory gating system. Daily front and lateral X-ray imaging was used for positioning. The PBT schedule was 33.0 Gy (RBE) in 15 fractions over 3 weeks in the combination therapy group and 72.6 Gy (RBE) in 33 fractions over 7 weeks in the PBT-only group. The PBT dose was modulated appropriately considering the response of the primary tumor as determined using endoscopy and PET-CT images. If the reduction in the maximal diameter of the primary tumor was < 50%, 1 to 3 fractions of PBT were performed without replanning. On the other hand, PBT was stopped without administering 1 to 3 fractions if the degree of tumor reduction was adequate and the patient had esophageal ulcer.

### Initial X-ray irradiation

Treatment planning for X-ray irradiation was also based on three-dimensional CT images taken at 2.5 mm intervals. The patients received PBT along with the initial X-ray irradiation (combination therapy) if they had ≥ T2 disease or lymph node metastases. However, patients were treated with PBT without initial X-ray irradiation if they had severe cardiopulmonary complications, such as interstitial pneumonitis or myocardial infarction, their performance status was 2 or they refused X-ray irradiation.

The cephalic and caudal borders of the initial X-ray irradiation fields included the bilateral supraclavicular nodes and cephalic plexus for thoracic or abdominal esophageal cancer. For cephalic esophageal cancer, we irradiated the region from the laryngopharynx to the carina. 10-MV X-ray irradiation was used with anteroposterior fields. The field number was generally two, whereas three fields were used for the field within a field technique when there was a large hot area. The daily X-ray irradiation fraction was 1.8 Gy, and the irradiation schedule was 36.0 Gy in 20 fractions delivered over 4 weeks.

### Evaluation and follow-up

All patients underwent endoscopy and PET to evaluate the initial tumor response within three months of the completion of treatment. The follow-up interval was every 2–3 months for the first year and every 3–6 months thereafter. Endoscopy, CT and PET-CT were performed if necessary.

Complete response was defined as the complete disappearance of all detectable tumors, partial response was defined as a ≥ 50% reduction in the maximal diameter of the tumor and stable disease was defined as no decreases or increases in the tumor diameter. Progressive disease was defined as enlargement of the primary tumor or the appearance of new lesions, including lymph node and distant metastases. Toxicities were evaluated using the Common Terminology Criteria for Adverse Events version 4.0.^19^

### Statistical analysis

The statistical tests were performed using the IBM SPSS Statistics version 22 software package (SPSS Inc., Chicago, IL, USA). The overall survival (OS) time was defined as the time between the start of treatment and the last follow-up. The local control time was defined as the time between the start of treatment and the date on which tumor recurrence was found or the last follow-up. The Kaplan–Meier method and log-rank test were applied to estimate survival probabilities and compare the survival rates, respectively.

## Results

### Patients

Twenty-six older patients were treated for esophageal cancer using PBT with or without initial X-ray irradiation between January 2009 and June 2013. All of these subjects were treated without any concurrent treatments, including chemotherapy. Of these 26 patients, 2 were excluded from the analysis because of distant metastasis and 4 were excluded for uncontrolled cancer at other sites. The characteristics of the remaining 20 patients, including 9 with inoperable cancer, are summarized in Table 1. All 20 patients completed their treatment. The cohort comprised 14 men and 6 women, with a median age of 78 years (range: 65–89 years). The median follow-up time was 26.5 months (range: 6–62 months). Comorbidities included interstitial pneumonitis owing to collagen disease (2 patients), chronic obstructive pulmonary disease (3 patients), myocardial infarction (5 patients), chronic heart failure (3 patients), chronic renal failure (2 patients) and diabetes mellitus (1 patient). Lymph node metastasis was present in 7 patients, and 10 patients had stage II/III cancer. Eleven patients were treated with PBT alone, and 9 patients were treated with combination therapy. The median dose of PBT was 72.6 Gy (RBE) (range: 66.0–74.8 Gy (RBE)) for PBT alone and 33.0 Gy (RBE) (range: 30.8–39.6 Gy (RBE)) for the combination therapy. With regard to the dose of X-ray irradiation, all patients received 36.0 Gy (RBE), except for one patient who received 32.4 Gy.

### Survival and local control

**FIGURE 2.**
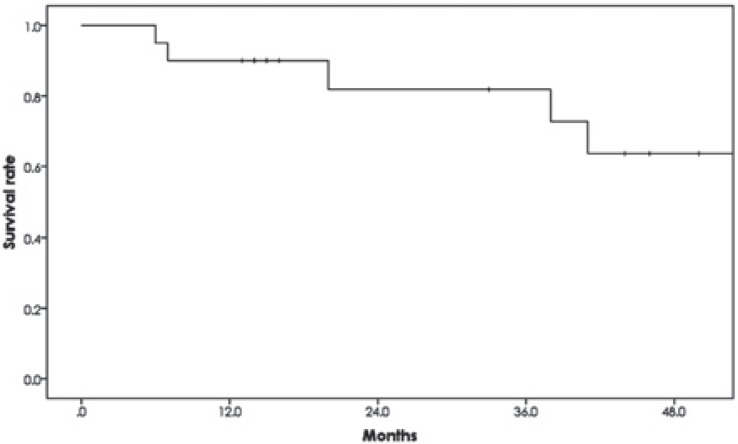
Overall survival rate of the patients with esophageal cancer after proton beam therapy. The 1- and 2-year overall survival rates were 90.0% and 81.8%, respectively.

**FIGURE 3.**
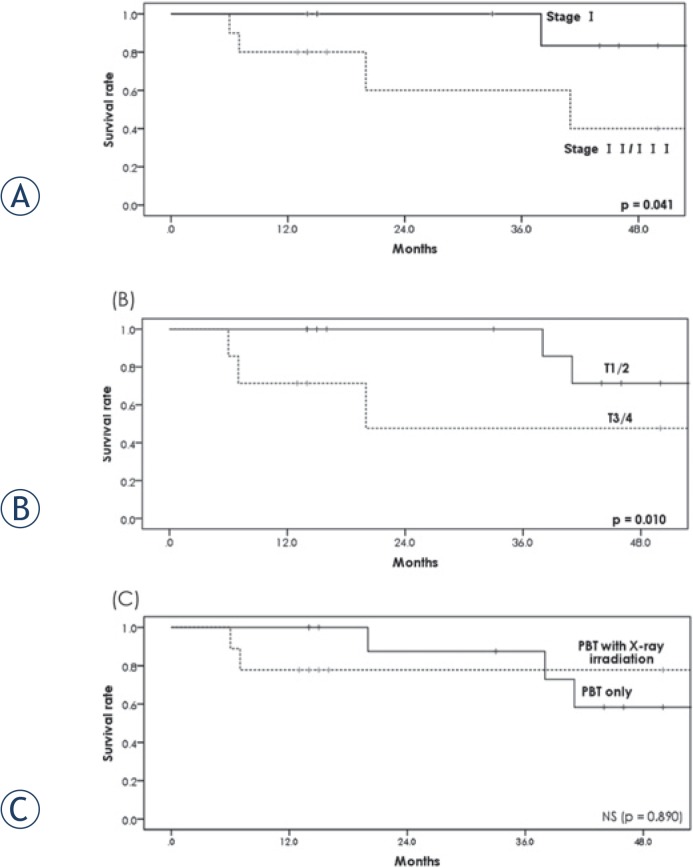
**(A)** Overall survival rate of the patients with stage I and II/III esophageal cancer. The 2-year overall survival rate was statistically different between the two groups (p = 0.041). **(B)** Overall survival rate of the patients in T 1/2 and T 3/4. The 2-year overall survival rate was statistically different between the two groups (p = 0.010). **(C)** Overall survival rate of the patients receiving proton beam therapy alone or proton beam therapy with initial X-ray irradiation. The 2-year overall survival rate was not statistically different between the two groups (p = 0.890). NS = not significant; PBT = proton beam therapy

**FIGURE 4.**
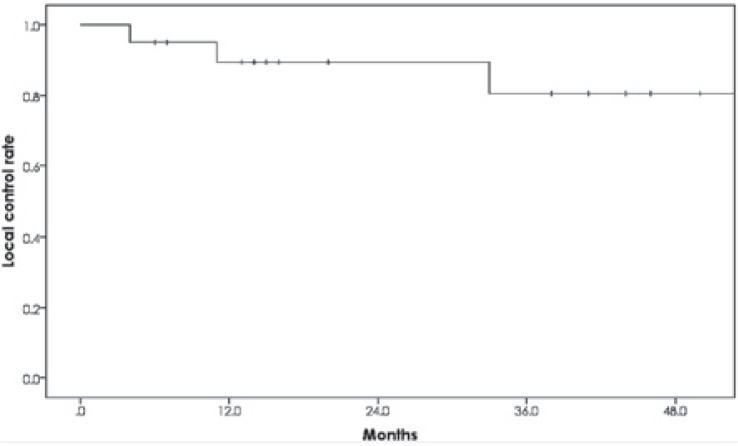
Local control rate for the patients with esophageal cancer after proton beam therapy. The 2-year local control rate was 89%.

All patients were followed for at least 13 months or until death. Six patients died, 4 from esophageal cancer and 2 from other causes (1 from bacterial pneumonia and 1 from another cancer). The 1- and 2-year OS rates were 90.0% (95% confidence interval [CI]: 76.9%–100%) and 81.8% (95% CI: 62.4%–100%), respectively (Figure 2). There was a significant difference in the 2-year OS rate between the patients with stage I (100%) and stage II/III (60.0%) cancers (p = 0.041) (Figure 3A). There was also a significant difference in the 2-year OS rate between the patients with T category 1/2 (100%) and T category 3/4 (47.6%) (p = 0.010) lesions (Figure 3B). On the other hand, the OS rate with or without initial X-ray irradiation was not significantly different (p = 0.890) (Figure 3C). Seventeen (85%) patients achieved a complete response and 3 (15%) achieved a partial response. Three patients (1 treated with PBT alone and 2 treated with the combination therapy) had local recurrence. The 2-year local control rate was 89.4% (95% CI: 75.5%–100%) (Figure 4).

### Failure patterns

Seven patients had recurrence. One patient had lymph node recurrence within the PBT field, 3 had distant metastases and 3 had local recurrence. There were no primary tumors or sites of lymph node recurrence outside the irradiation field in the PBT-only group.

### Toxicities

There were no grade 4 or 5 toxicities after treatment (Table 2). Of the 20 patients, 6 (30%) had grade 2 esophageal ulcers, 2 (10%) had grade 2 pneumonitis and 2 (10%) had grade 2 pleural effusion. One patient (5%) with an esophageal ulcer required intravenous hyperalimentation (grade 3 esophageal ulcer), and the ulcer healed one month later. Two patients (10%) with esophageal stenosis were treated with dilation using endoscopy (grade 2 esophageal stenosis). One of these patients developed an esophageal fistula (grade 2 esophageal fistula) just after dilation and was treated with the insertion of a stent in the esophagus. Neither patient required surgery. One patient (5%) with pneumonitis was treated with oxygenation and steroid administration two years after PBT because of dyspnea (grade 3 pneumonitis). In that case, the dyspnea was relieved 3 days later, and the dose of steroids was gradually reduced.

## Discussion

We herein demonstrated that PBT without chemotherapy is efficacious and safe for the treatment of older patients with esophageal cancer. To the best of our knowledge, this is the first report on the use of PBT without chemotherapy in older patients with esophageal cancer.

Radiotherapy alone is one choice for treating older patients with esophageal cancer who cannot receive CRT or surgery. Kawashima *et al.*^10^ reported the results of 66 Gy X-ray irradiation without chemotherapy in 51 older patients with no lymph node metastasis: the 1- and 2-year OS rates were 71% and 53%, respectively. Additionally, Cooper *et al.*^3^ reported 1- and 2-year OS rates after 64 Gy radiotherapy alone of 34% and 10%, respectively, Smith *et al.*^9^ reported 1-year and 2-year OS rates for older esophageal cancer patients treated with X-ray irradiation alone of 16% and 7%, respectively and Nemoto *et al.*^20^ reported 1- and 2-year OS rates after radiotherapy alone (median total dose: 65.5 Gy) for superficial esophageal cancer (stage I) of 88% and 73%, respectively. Our results, including those for the 7 patients with lymph node metastases, showed superior 1- and 2-year OS rates to those observed in these studies (Table 3). These results may differ because we were able to administer higher CTV doses with less exposure to organs at risk, such as the lungs and heart, although a previous report indicated that higher doses do not improve outcomes in cases of CRT.^4^

Studies of esophageal cancer treated with CRT have reported 5-year OS rates of 11%–75.7%; these cohorts included older patients.^3^,^5^,^6^,^8^ In a comparative study of CRT versus surgery alone for the treatment of esophageal cancer, Ariga *et al.*^5^ reported a 3-year OS rate of 69.1% for CRT patients in stage II/III and 47.9% for surgery patients in stage II/III; for patients with stage I cancer, the 2-year OS rate was 100% in the CRT group and 90% in the surgery alone group. Ishikura *et al.*^7^ reported long-term toxicities after CRT in a study of 139 patients, with grade 4 or 5 esophagitis (7 patients), pneumonitis (4 patients) and pericardial effusion (1 patient). Our results showed an equivalent 2-year OS rate for patients with stage I cancer but an inferior OS rate for patients with stage II/III cancer. This result suggests that PBT without chemotherapy is sufficient for treating stage I esophageal cancer, although patients with stage II/III esophageal cancer have a higher OS when treated with concomitant chemotherapy. However, older patients receiving platinum-based chemotherapy develop significantly more grade 3–5 toxicities than younger patients.^21^,^22^ In addition, patients treated with CRT for esophageal cancer experience more life-threatening acute toxicities than those treated with radiotherapy alone (CRT: 8%; radiotherapy alone: 2%).^3^ Higher grade toxicities are more common in patients receiving concomitant CRT, particularly older patients. Therefore, the administration of concomitant chemotherapy is not possible in all older patients; PBT without chemotherapy may be a feasible treatment choice for older patients with stage II/III esophageal cancer, particularly for older patients who have cardiopulmonary comorbidities, renal failure or a bad performance status.

No broad consensus has been established regarding the optimal CTV protocol for elective nodal irradiation in cases of esophageal cancer. Zhao *et al.*^23^ evaluated the results of late-course accelerated hyperfractionated involved-field conformal radiotherapy for locally advanced esophageal squamous cell carcinoma, reporting OS rates of 77% at 1 year and 56% at 2 years, although both T4 and N1 patients were included. In addition, the rate of out-field node recurrence alone was only 8%. Similarly, Kawaguchi *et al.*^24^ observed a rate of out-field lymph node recurrence alone of 11%. Ji *et al.*^25^ reported that lymph nodes located near esophageal cancer lesions receive considerable incidental doses of irradiation to the involved field, which may eliminate subclinical lesions. Zhang *et al.*^26^ reported the results of involved-field irradiation for esophageal cancer, including patients with lymph node metastasis (73.4%); they observed that the rate of out-field lymph node recurrence was as high as 30%. In our study, we observed no recurrence outside the irradiation field in patients receiving PBT alone and found no significant differences in the OS rates between the patients treated with PBT alone and those treated with the combination therapy. These results suggest that involved-field irradiation is a sufficient treatment for esophageal cancer without lymph node metastasis. Furthermore, PBT has advantages over other treatments for esophageal cancer because higher radiation doses can be administered without increasing the toxicity.

High radiation doses reportedly do not improve the OS rate in cases of CRT^4^; however, the optimal dose for radiotherapy alone has not been determined. The OS rate may increase if patients receive a higher dose of radiation. In Japan, even older patients receive 66 Gy for radiotherapy alone^10^; most patients receiving PBT can tolerate doses higher than 66 Gy (RBE). In the current study, our patients who underwent PBT experienced no severe or fatal toxicities within the follow-up period, although they received doses higher than 66 Gy. There were 2 patients who had esophageal stenosis in the current study, however, the esophageal stenosis was severe in both cases because of esophageal cancer before starting treatment and the stenosis remained even after they achieved a complete response. Kawashima *et al.*^10^ reported that 3 (5.9%) patients receiving 66 Gy X-ray irradiation developed grade 5 pneumonitis within 90 days of the start of radiotherapy. Mizumoto *et al.*^15^ reported the results of PBT (median total dose of combined X-ray and proton beam: 80.0 Gy (RBE); median dose of PBT alone: 79.0 Gy (RBE) without chemotherapy for locally advanced T1-4 N0/1 M0 esophageal cancer. The only toxicity observed was non-healing ulcers in 4 (8%) patients. These results suggest that, compared with X-ray therapy alone (dose: > 60 Gy (RBE)) or PBT (dose: > 80 Gy (RBE)), PBT is a safe and feasible treatment for esophageal cancer when the dose is 66.0 to 75.6 Gy (RBE).

We used initial X-ray irradiation for elective nodal irradiation, because the available field size of PBT at our institute is 15 cm × 15 cm. Some researchers have also reported using combination therapy.^14^,^15^ When the patients received the initial X-ray irradiation at our institute, PBT was performed as shown in Figure 1. Although the proton beam was stopped when it reached a location close to the spinal cord (Figure 1A), the irradiation dose for the spinal cord was adequately reduced and no patients with radiation myelopathy were observed as of the last follow-up. On the other hand, the lung regions received a high dose (Figure 1B), however, the irradiation dose for the lung of PBT was less than the oblique opposed X-ray irradiation following anteroposterior irradiation for elective nodal irradiation. As a result, we think that combination therapy is therefore a practical and safe technique for treatment with esophageal cancer.

There are two limitations associated with this study. First, the number of patients was very small and we only included patients from a single institution. However, the current study revealed the high 1- and 2-year OS rates with following survivors for at least 13 months. Second, the follow-up time was short, as we started using PBT only in 2008. Therefore, longer follow-up is needed to ascertain the long-term OS rate and toxicities.

The high 1- and 2-year OS rates with acceptable toxicity observed in this study indicated that high-dose 66.0–75.6 Gy (RBE) PBT without chemotherapy was an efficacious and safe treatment for older patients with esophageal cancer.

## Figures and Tables

**TABLE 1.** Patient characteristics

**Characteristics**	**Patients**
Age (years)	
Median	78
Range	65–89
65–69	2 (10%)
70–74	5 (25%)
75–79	6 (30%)
80–89	7 (35%)
Gender	
Male	14 (70%)
Female	6 (30%)
Performance status	
0	7 (35%)
1	11 (55%)
2	2 (10%)
Follow up time (months)	
Median	26.5
Range	6–62
T category^*^	
T1	8 (40%)
T2	5 (25%)
T3	6 (30%)
T4	1 (5%)
N category^*^	
N0	13 (65%)
N1	4 (20%)
N2	3 (15%)
Stage^*^	
I	10 (50%)
II	5 (25%)
III	5 (25%)
Tumor location	
Cervical	3 (15%)
Upper thoracic	4 (15%)
Mid thoracic	9 (45%)
Lower thoracic	4 (25%)
Histopathology	
Squamous cell	
carcinoma	19 (95%)
Adenocarcinoma	1 (5%)
Proton dose in PBT with initial X-ray irradiation (n=9) (Gy (RBE))	
Median	33.0 (total dose: 69.0)
Range	30.8–39.6 (total dose: 66.8–75.6)
Proton dose in PBT alone (n=11) (Gy (RBE))	
Median	72.6
Range	66.0–74.8

PBT = proton beam therapy; RBE = relative biological dose effectiveness;

* Numbers correspond to the tumor-node-metastasis system of classification (International Union Against Cancer criteria)

**TABLE 2.** Toxicities

**Toxicities**	**Grade 0**	**Grade 1**	**Grade 2**	**Grade 3**	**Grade 4**	**Grade 5**
**Esophagitis**	3 (15%)	3 (15%)	14 (70%)	0	0	0
**Esophageal ulcer**	13 (65%)	0	6 (30%)	1 (5%)	0	0
**Esophageal stenosis**	18 (90%)	0	2 (10%)	0	0	0
**Esophageal fistula**	19 (95%)	0	1 (5%)	0	0	0
**Pneumonitis**	6 (30%)	11 (55%)	2 (10%)	1 (5%)	0	0
**Pleural effusion**	12 (60%)	6 (30%)	2 (10%)	0	0	0
**Pericardial effusion**	17 (85%)	3 (15%)	0	0	0	0

**TABLE 3.** Previous results of radiation therapy without chemotherapy for esophageal cancer and our result

	**number of patients**	**T category**	**N category**	**median total dose**	**1-year OS**	**2-year OS**
**Nemoto *et al*., 2000^20^**	78	T1	N0	65.5 Gy	88%	73%
**Kawashima *et al*., 2006^10^**	51	T1–3	N0	66 Gy	71%	53%
**Cooper *et al*., 1999^3^**	62	T1–3	N0–1	64 Gy	34%	10%
**Smith *et al*., 2009^9^**	623	T1–4	N0–1	none	16%	7%
**Oto *et al*., 2015**	20	T1–4	N0–2	69.0 Gy (RBE) (PBT with X-ray) 72.6 Gy (RBE) (PBT only)	90%	81.8%

OS = overall survival; PBT = proton beam therapy; RBE = relative biological dose effectiveness
